# Economic Analysis of Vaccination Strategies for PRRS Control

**DOI:** 10.1371/journal.pone.0144265

**Published:** 2015-12-16

**Authors:** Daniel C. L. Linhares, Clayton Johnson, Robert B. Morrison

**Affiliations:** 1 Veterinary Diagnostic and Production Animal Medicine Department, College of Veterinary Medicine, Iowa State University, Ames, Iowa, United States of America; 2 Swine Health Department, The Maschhoffs LLC, Carlyle, Illinois, United States of America; 3 Department of Veterinary Population Medicine, College of Veterinary Medicine, University of Minnesota, Saint Paul, Minnesota, United States of America; University of Hong Kong, CHINA

## Abstract

Porcine reproductive and respiratory syndrome virus (PRRSv) is a swine-specific pathogen that causes significant increases in production costs. When a breeding herd becomes infected, in an attempt to hasten control and elimination of PRRSv, some veterinarians have adopted a strategy called load-close-expose which consists of interrupting replacement pig introductions into the herd for several weeks (herd closure) and exposing the whole herd to a replicating PRRSv to boost herd immunity. Either modified-live virus (MLV) vaccine or live field-virus inoculation (FVI) is used. This study consisted of partial budget analyses to compare MLV to FVI as the exposure method of load-close-expose program to control and eliminate PRRSv from infected breeding herds, and secondly to estimate benefit / cost of vaccinating sow herds preventatively. Under the assumptions used in this study, MLV held economic advantage over FVI. However, sensitivity analysis revealed that decreasing margin over variable costs below $ 47.32, or increasing PRRSv-attributed cost above $18.89 or achieving time-to-stability before 25 weeks resulted in advantage of FVI over MLV. Preventive vaccination of sow herds was beneficial when the frequency of PRRSv infection was at least every 2.1 years. The economics of preventative vaccination was minimally affected by cost attributed to field-type PRRSv infection on growing pigs or by the breeding herd productivity level. The models developed and described in this paper provide valuable tools to assist veterinarians in their efforts to control PRRSv.

## Introduction

Porcine reproductive and respiratory syndrome virus (PRRSv) causes significant production losses which result in substantial increases in production costs [1–3]. Thus, control strategies are commonly employed to decrease the production losses.

Experimentally, vaccination with modified live PRRS virus decreases reproductive loss after infection and therefore, many veterinarians recommend preventatively vaccinating sow herds in case of infection with field virus. Vaccination has a cost however, and not all vaccinated herds become infected. Therefore, there is a need to estimate the benefit / cost of preventatively vaccinating the sow herd.

When a sow herd becomes infected, in an attempt to hasten control and elimination of PRRSv from the breeding herd, some veterinarians have adopted a strategy called load-close-expose which consists of interrupting replacement gilt introductions into the herd for several months (herd closure) and exposing the whole herd to a replicating PRRSv. Either modified-live virus (MLV) vaccine or field-virus inoculation (FVI) is used [4–6]. Herds that used MLV required 7 additional weeks to reach PRRSv-stability compared to herds that used FVI [7]. However, MLV herds recovered production levels 11 weeks sooner and had less total loss in pigs weaned (advantage of 1,443 pigs per 1,000 sows) [7]. Depending on economic conditions at the time, there is a need to determine which program (MLV or FVI) has better overall economic advantage for a farm.

Partial budgeting is a method commonly used in veterinary medicine to determine the economic benefit of interventions [8–11]. Basically, partial budget analysis takes into account: a) increase in income, b) reduction or elimination of income, c) reduction or elimination of costs and, d) increase in costs [12].

This study consisted of partial budget analyses to compare MLV to FVI as the whole herd exposure method of load-close-expose program to control and eliminate PRRSv from infected breeding herds, and secondly to estimate benefit / cost of vaccinating sow herds preventatively.

## Methods

Three partial budget models were created. The first model was a deterministic simulation (model A) of net margin over feed costs (MOFC) for a breeding herd of 1,000 sows comparing MLV vs. FVI as the exposure method of a load-close-expose program to control and eliminate a field-type PRRSv infection from a pig production system. The second approach was a stochastic model using Monte Carlo simulation to compare MLV and FVI programs (model B). The third model estimated the benefit / cost of vaccinating sows herds preventatively (model C).

### Model A

#### Input variables

Outcome variables from the literature and from our previous work [7] were used as input variables for this economic model (described in Table 1).

**Table 1:** Input variables for the partial budget model that compared economic outcomes of MLV herds compared to FVI herds in load-close-expose programs to produce PRRSv-negative pigs from infected breeding herds.

Variable	Meaning in the model	Values and source
**Time to PRRSv stability (TTS)**	As TTS increases, the number of batches of PRRSv-positive pigs weaned increases, impacting the cost of growing pig production	FVI and MLV median TTS, 25 and 32 weeks respectively (Linhares et al., 2014).
**Time to baseline production (TTBP)**	Used to adjust number of pigs weaned before TTS was reached	FVI and MLV median TTBP, 10 and 21 weeks respectively (Linhares et al., 2014).
**Total loss (pigs not weaned/1,000 sows)**	Pigs not weaned represent decreased revenue	FVI and MLV average total loss, 1,222 and 2,665 pigs respectively (Linhares et al., 2014)
**Cost of PRRSv-positive pig at weaning**	PRRSv-positive pigs have extra cost of production	$ 13.52 [1]. Also, variability was added in the model considering the following values $ 10, 15 and 20.
**Margin over variable cost (MOVC)** *	Pigs not weaned represent opportunity margin	$66.72 (Morrison, personal information 2012)
**Number of pigs weaned per sow per year**	Used to calculate productivity level of a sow farm	26.00 pigs weaned/sow/year

* Briefly, it was considered 12 months average of future prices of feed, plus extra variable costs including veterinary services ($4/pig) and trucking ($2/pig).

#### Calculations

Additional Costs—MLV vaccine was estimated to cost $1.00 per dose with 3 doses being administered per sow per year. For FVI, $100 was assumed for extra diagnostic costs to test the PRRSv inoculum used for herd exposure.

Decreased costs–None.

Additional revenue–None.

Decreased revenue–Two sources of decreased were considered. First, the opportunity cost for pigs not weaned was estimated by margin over cost of feed not consumed. Market price of $80.00/cwt carcass, average carcass weight of 200 lbs, and wean to finish feed cost of $ 99.49 per pig were assumed to yield an opportunity cost of $66.72 per pig not weaned.

Secondly, the opportunity cost for impeded growing performance was calculated by multiplying the number of weeks it took for herds to achieve “time to PRRS stability” status (TTS) by the “number of pigs weaned per week” (500 pigs) and “cost attributed to PRRSv infection” in growing pigs ($13.52) [1]. As previously described, TTS was achieved when obtained 4 consecutive negative PCRs for PRRSv RNA in a monthly basis testing of at least 30 piglet blood samples [7]. Time to baseline production (TTBP) was defined as time, in weeks, it took to recover the levels of ‘weaned pigs per week’ that the herd had prior to PRRSv-detection (i.e. time to “in control” levels of productivity). To avoid double counting, “pigs not weaned” from “TTBP” to “TTS” was subtracted from the calculation, resulting in the formula listed below:

Opportunitycostforimpededgorwingpermoformance=((TTS−TTBP)*pigsweanedperweek*$13.52)+((TTBP*pigsweanedperweek−pigsnotweanedper1,000sows)*13.52)

The overall opportunity cost was obtained by adding the opportunity costs for pigs not weaned and for reduced growing performance. Then, the opportunity cost for farms that were treated with FVI was subtracted from that of farms that used MLV to show the advantage (or disadvantage) to use MLV instead of FVI.

To describe the relative importance of key variables of the model, sensitivity analyses were performed. The first sensitivity analysis considered differences in the ‘extra production cost’ attributable to PRRSv-infection and pig margin over variable cost. The second sensitivity analysis considered weeks for TTS for MLV herds that allowed the economic model to converge at a break-even point with FVI herds.

### Model B

#### Input variables and calculations

The same input variables used in the model A were used in model B. However, model B took into account variability in TTS, TTBP and total loss parameters (Table 2) using Monte Carlo simulation approach [13]. Kolmogorov-Smirnov test indicated that the TTS, TTBP and total loss parameters fitted Normal distributions at alpha level of 0.05, and therefore, the distributions assumed for these 3 parameters were Normal [7]. The calculations of opportunity costs were performed using the same formulas described in model A. However, because model B is stochastic, the outcome was a distribution that took 3,000 random samples (iterations) from the TTS, TTBP and total loss distributions. To execute the model B, the RiskSim ® v 2.43 software (TreePlan Inc, San Francisco, CA) was used.

**Table 2:** Input variables for the stochastic simulation (model B).

Variable	Distribution	Distribution parameters
**Time to PRRSv stability (TTS)**	Normal	FVI herds: mean 25.02, Std. Dev 6.91
		MLV herds: mean 31.87, Std. Dev 8.84
**Time to baseline production (TTBP)**	Normal	FVI herds: mean 18.28, Std. Dev 7.56
		MLV herds: mean 8.48, Std. Dev 7.18.
**Total loss (pigs not weaned/1,000 sows)**	Normal	FVI herds: mean 2,665, Std. Dev 1,980
		MLV herds: mean 1,222 Std. Dev 1,677
**Cost of PRRSv-positive pig at weaning**	Fixed (no distribution)	$ 13.52 [1]. Also, variability was added in the model considering the following values $ 10, 15 and 20.
**Margin over variable cost (MOVC)**	Fixed (no distribution)	$66.72 (Morrison, personal information 2012)
**Number of pigs weaned per sows per year**	Fixed (no distribution)	26.00 pigs weaned/sow/year

### Model C

The same input variables used in the model A were used in model C. The “preventative vaccination value” model has 3 components:

1. **Estimation of long term benefit of preventative vaccination with MLV PRRSv vaccine, given that a breeding herd eventually becomes infected with field-type PRRSv.** Herds with preventative vaccination achieved TTBP and TTS 10 weeks and 3.3 weeks sooner than herds that did not practice preventative vaccination (11 weeks versus 21 weeks and 26.0 versus 29.3 weeks, respectively) [7]. Similarly herds with prior immunity (those practicing preventative vaccination) had 550 piglets not weaned/1000 sows after infection with field-type PRRSv, while PRRSv-negative herds (not vaccinating) lost 2,457 pigs not weaned/1000 (Table 3) [7].
2. **Quantification of impact on production performance of systems infected with attenuated PRRSv, compared to those not infected with any type of PRRSv.** A breeding herd that was continuously vaccinated with PRRSv-MLV was estimated to have decreased annualized production of 2 pigs per sow per year [14, 15], which resulted in an increase of $2.96 dollars per weaned pig (worst case scenario). Also, it was considered that growing pig flows positive for a MLV PRRSv had an impact of $1.33 per marketed pig ($ 0.33 of average daily gain, $ 0.40 of feed conversion and $ 0.60 of other costs) compared to growing pig flows free of PRRSv.
3. **Estimation of wild type PRRSv infection frequency to justify need for preventative vaccination with attenuated PRRSv.** The outcome of the model was break-even frequency, in years, that a breeding herd would become infected with field type-PRRSv to justify preventative MLV-PRRSv practice. A sensitivity analysis was performed to compare the effects of ‘margin over feed per pig’ (MOFC), ‘sow herd productivity’ (measured as number of pigs weaned/sow/year) and ‘attributed PRRSv-cost per infected growing pig’ on the economic advantage of practicing preventative vaccination using MLV-PRRSv.

**Table 3:** Partial budget model to compare economic benefit of eliminating field type-PRRSv using load-close-expose methods from a herd with prior immunity (i.e. practiced preventative vaccination) over a PRRSv-negative 1,000 sows breeding herd.

Treatment	Cost to expose		Opportunity Cost for pigs not weaned				Opportunity Cost for w-f performance			
	Exposure		TTBP	Total loss	OC*		TTS	OC		Total OC
**Preventative vaccination**	$3,000	+	11	550	$36,696	+	26.0	$178,464	=	$218,160
**PRRSv-negative**	$100	+	21	2,457	$163,931	+	29.3	$176,276	=	$340,207
**Difference (Preventative-Free)**				** **	**$127,235**	** **	** **	**($2,188)**	** **	**$122,047**

* Opportunity costs

## Results and Discussion

Using the economic assumptions listed above, the longer TTS observed in herds exposed with MLV resulted in approximately $ 67,000 disadvantage per 1,000 sows compared to FVI herds. Conversely, the lower total production loss in MLV herds resulted in advantage of approximately $ 96,000 per 1,000 sows compared to FVI herds. The model A was built to combine the TTS and ‘total production loss’ parameters, and showed that a net advantage for MLV herds of $ 26,548 per 1,000 sows (Table 4).

**Table 4:** Partial budget model to compare economic benefit of MLV over FVI on a load-close-expose program to eliminate PRRSv from an infected 1,000 sows breeding herd.

Treatment	Cost to expose		Opportunity Cost for pigs not weaned				Opportunity Cost for w-f performance			
	Exposure		TTBP	Total loss	OC*		TTS	OC		Total OC
**MLV**	$3,000	+	12	1,222	$81,532	+	32	$199,799	=	$284,330
**FVI**	$100	+	20	2,665	$177,809	+	25	$132,969	=	$310,878
**Difference (MLV-FVI)**				** **	**$ 96,277**	** **	** **	**$-66,829**	** **	**$26,548**

* OC = Opportunity cost

**Fig 1:**
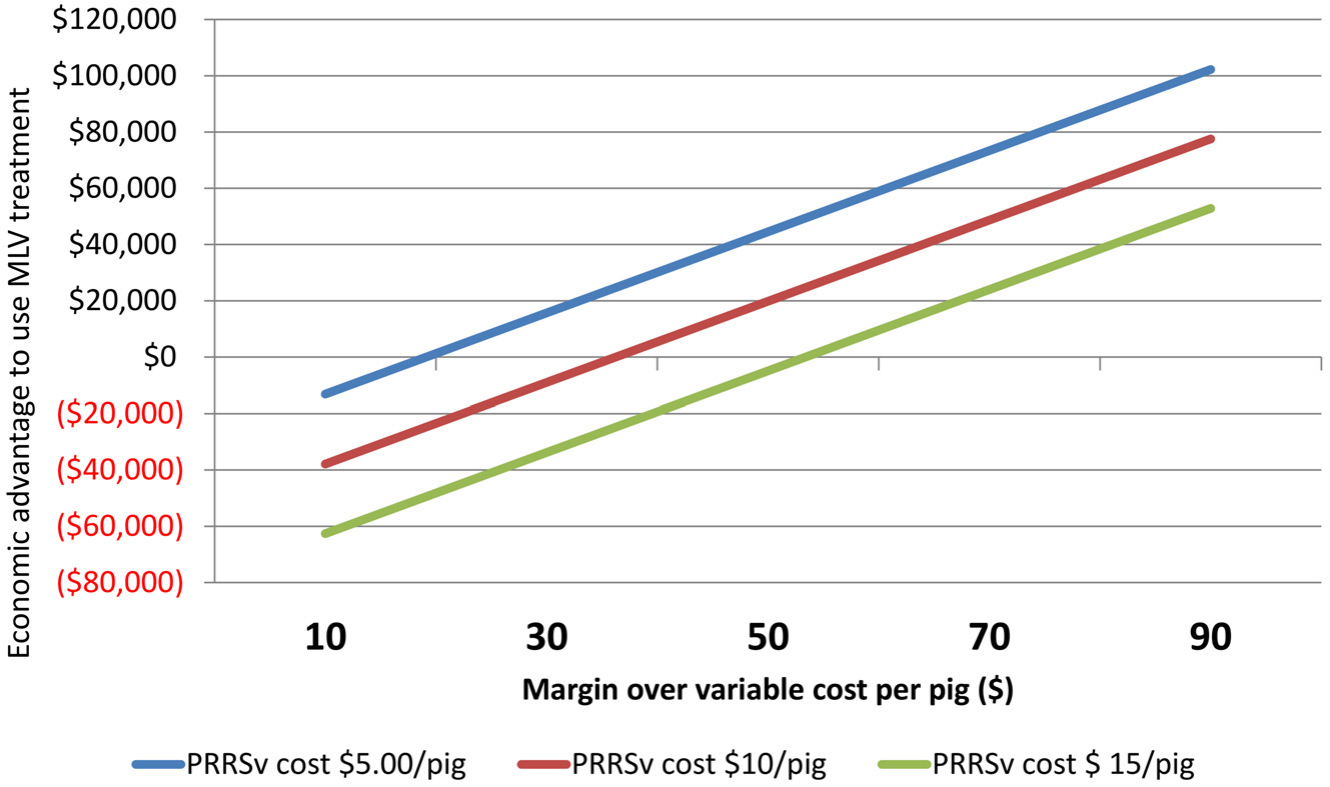
Impact of margin over variable cost (MOVC) and production cost attributable to PRRSv on advantage to use MLV.

Cost attributable to PRRSv represents extra production costs that PRRSv infected pigs have due to higher mortality, higher feed conversion rates and slower growing performance. The major contributor to variable costs in pig production is feed [16]. Besides feeding, health interventions and trucking also contribute to MOVC. We constructed a sensitivity analysis to describe the influence of ‘MOVC per pig’ and ‘added pig production cost attributed to PRRSv” on the advantage to use MLV compared to FVI as whole herd exposure method (Fig 1). These two parameters (MOVC and PRRSv-attributed cost) influenced the break-even between FVI and MLV programs in opposite directions. More specifically, increasing MOVC resulted in increase of the MLV-program advantage, whereas increasing PRRSv-attributed cost of production increased the FVI-program advantage. Is important to remind that MOVC changes based on crop and pork market conditions, while PRRSv-attributed cost on pig production varies based on factors including specific PRRSv-strain virulence, level of herd immunity, presence of co-infections and environmental (stress) conditions.

**Fig 2:**
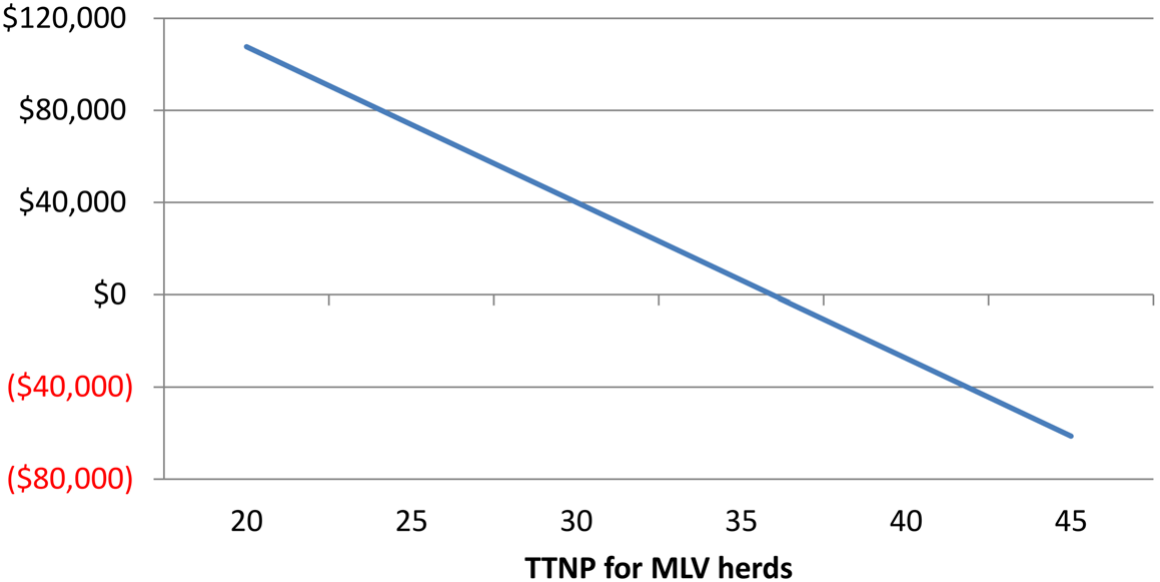
Break-even analysis between FVI and MLV varying TTS for MLV, considering TTNP for LVI as 25 weeks.

**Fig 3:**
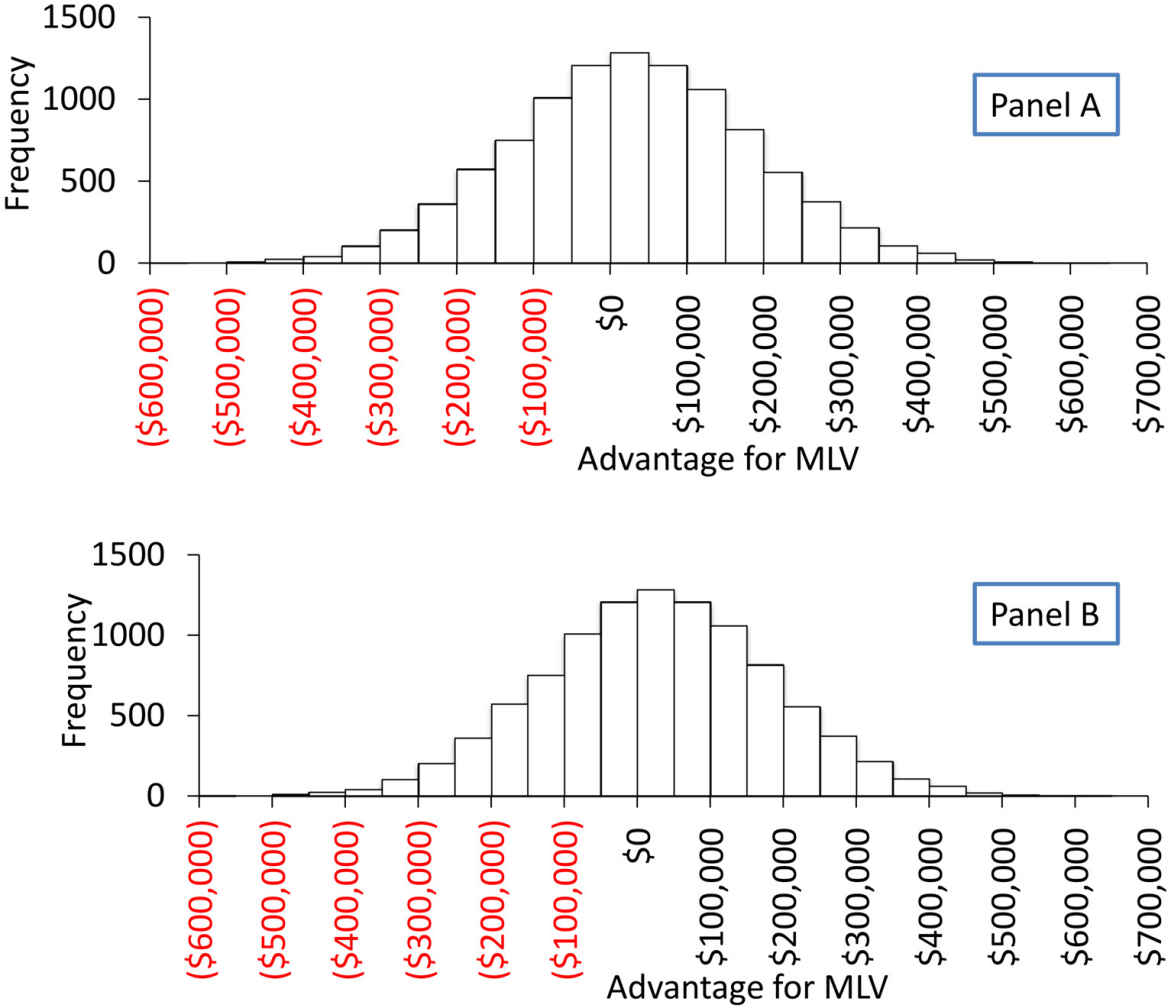
Distribution of economic advantage of farms that used MLV in comparison to those that used LVI as part of load-close-expose program. Outcome of model B, a Monte Carlo simulation of economic advantage of MLV compared to LVI. (A) is the outcome illustrated as a probability density function. (B) is the outcome illustrated as cumulative density function.

Another important factor that influenced the outcome of model A was time to achieve stability (TTS). Sensitivity analysis demonstrated that MLV held economic advantage over FVI when TTS was below 38 weeks (Figs 1 and 2). Incorporating variability for the distributions of TTS, TTBP and total loss (Fig 3) suggested an economic benefit for MLV for approximately 60% of the iterations.

Overall, the sensitivity analyses revealed that decreasing MOVC below $ 47.32, or increasing PRRSv-attributed cost above $18.89 or achieving TTS before 25 weeks resulted in advantage of FVI over MLV. Limitations of models A and B lie in the assumption that all herds that reached TTS would have truly eliminated PRRSv. It has been reported that PRRSv can still be circulating in a low proportion of herds that were incorrectly considered stable [17]. It was also assumed that growing pig sites were operated all in–all out on a weekly basis. Moreover, it was considered that in MLV herds, there was virulent PRRSv circulating in the herd and causing decreased performance until TTS was reached. Model A was a univariate deterministic model and did not take into account covariates such as prior infection status for PRRSv or days from infection to intervention.

Preventive vaccination against PRRSv has been considered as strategy to minimize PRRS-associated losses when pig populations become infected with wild type virus [18, 19]. In fact, it has been shown that breeding herds with recent history of PRRSv infection (i.e. prior PRRSv-immunity) achieved stability sooner and had less production impact than those without history of PRRSv infection (i.e. no PRRSv herd immunity) [17]. However, immunizing breeding herds using attenuated PRRSv results in increased cost of production due to the commercial vaccine cost and also due to potential negative impact of the attenuated replicating virus on productivity levels [14, 15, 20, 21].

**Fig 4:**
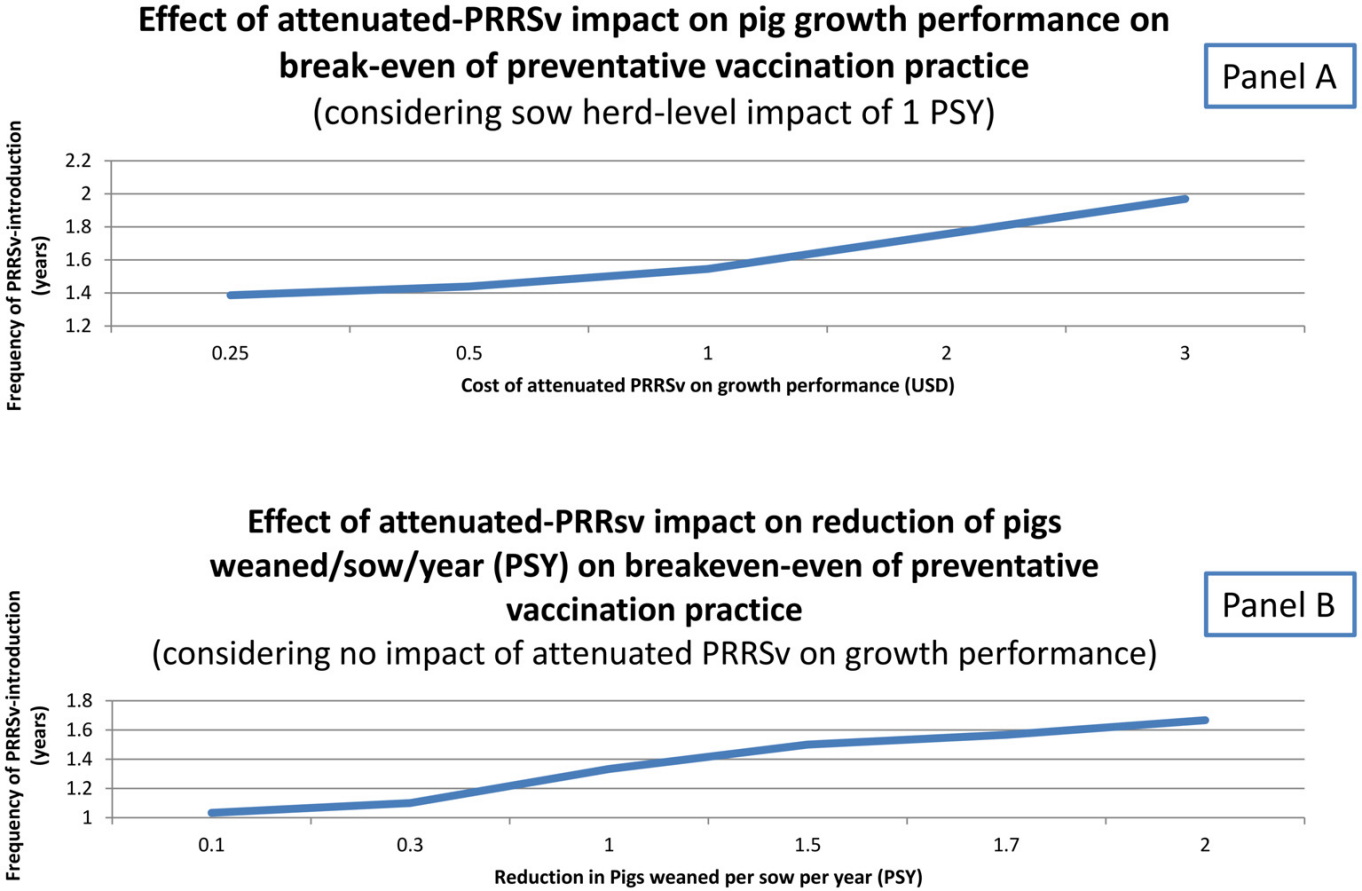
Break-even analysis of preventative vaccination practice according to cost of attenuated-PRRSv on growth performance or magnitude of reduction on pigs/sow/year (PSY) due to attenuated PRRSv. (A). Effect of attenuated-PRRSv impact on pig growth performance on break-even of preventative vaccination, considering sow herd-level impact of 1 PSY. (B) Effect of attenuated-PRRSv impact on reduction of pigs weaned/sow/year on break-even of preventative vaccination, assuming no impact of attenuated PRRSv on growth performance.

**Fig 5:**
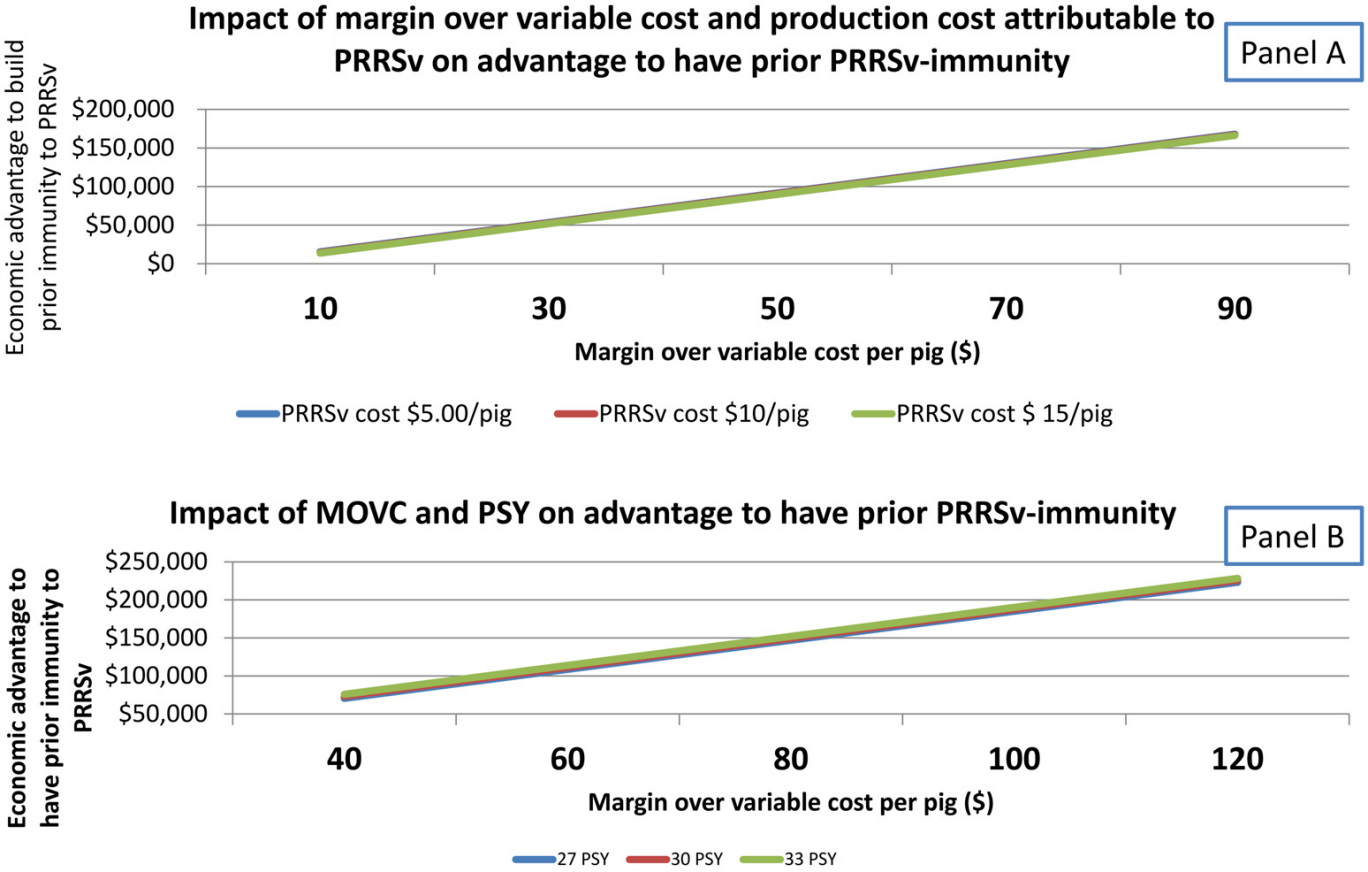
Break-even analysis of preventive vaccination practice according to cost of attenuated-PRRSv on growth performance or magnitude of reduction on pigs/sow/year (PSY) due to attenuated PRRSv. (A) Impact of margin over variable cost (MOVC) and production cost attributable to PRRSv on advantage to have prior PRRSv-immunity. (B) Impact of MOVC and PSY on advantage to have prior PRRSv-immunity.

Thus, preventive vaccination of sow herds can be beneficial depending on the risk (i.e. expected frequency) of infection with wild type PRRSv (Table 5). The potential impact of MLV on sow and pig performance plays a critical role in this decision and is poorly understood. This study provided models on the effect of key factors (PRRSv-attributed cost on pig production, MOVC, impact of MLV on number of pigs produced per sow per year, impact of MLV on growth performance) on the economic benefit of practicing preventive vaccination (Figs 4 and 5). Sensitivity analyses showed that ‘cost of attenuated PRRSv on growth performance’ and ‘reduction in pigs per sow per year (PSY)’ influenced the break even (number of years between PRRSv outbreaks) to justify preventive vaccination. Lower attenuated-PRRSv-impact on productivity (via growth performance or breeding herd productivity) reduced the break even for practicing preventive vaccination. Considering 1.5 pigs and $1.00 for reduction in PSY and attenuated PRRSv-impact on growth performance respectively, the break even for preventative vaccination was 1 year and 9 months. In other words, when a given breeding herd has a frequency of wild type PRRSv outbreak shorter than 1 year and 9 months it is economically worth it practicing preventative vaccination according to the assumptions of Model C.

**Table 5:** Minimum infection frequency (in years) to justify PRRSv preventative vaccination with attenuated vaccine on a production system with one thousand sows.

	Benefit over cost (USD)		Probability of field-type PRRSv introduction per year (Pct)*		Project value (USD)*
**Breeding herd becomes infected with field-type PRRSv**	122,047	*	0.49	=	59,447
**Breeding herd does not infect with field-type PRRSv**	(115,899)	*	0.51	=	(59,447)
**Long term difference**					$ 0
**Minimum frequency of field type-PRRSv-infection to justify preventative vaccination with MLV-PRRSv**					2.1 years

* Probability of production system infecting with field-type PRRSv to reach break-even of benefit of preventative vaccination.

Sensitivity analysis is an important tool for considering the effects of influential variables in the decision to be made. Partial budget models are useful for understanding the economic implications of production costs and losses. The models developed and described in this paper provide valuable tools to assist veterinarians in their efforts to control PRRS virus.
