# Correction: Economic Analysis of Vaccination Strategies for PRRS Control

**DOI:** 10.1371/journal.pone.0150444

**Published:** 2016-04-18

**Authors:** Daniel C. L. Linhares, Clayton Johnson, Robert B. Morrison

In the title of the article, the word “Vaccination” should be “Immunization.” The correct title is: Economic Analysis of Immunization Strategies for PRRS Control. The correct citation is: Linhares DCL, Johnson C, Morrison RB (2015) Economic Analysis of Immunization Strategies for PRRS Control. PLoS ONE 10(12): e0144265. doi:10.1371/journal.pone.0144265

There is an error in the seventh sentence of the Abstract. The correct sentence is: Preventive vaccination of sow herds was beneficial when the frequency of PRRSv infection was at least every 1 year and 9 months.

There are errors in Figs 3–5. Please see the corrected Figs 3–5 below.

**Fig 3:**
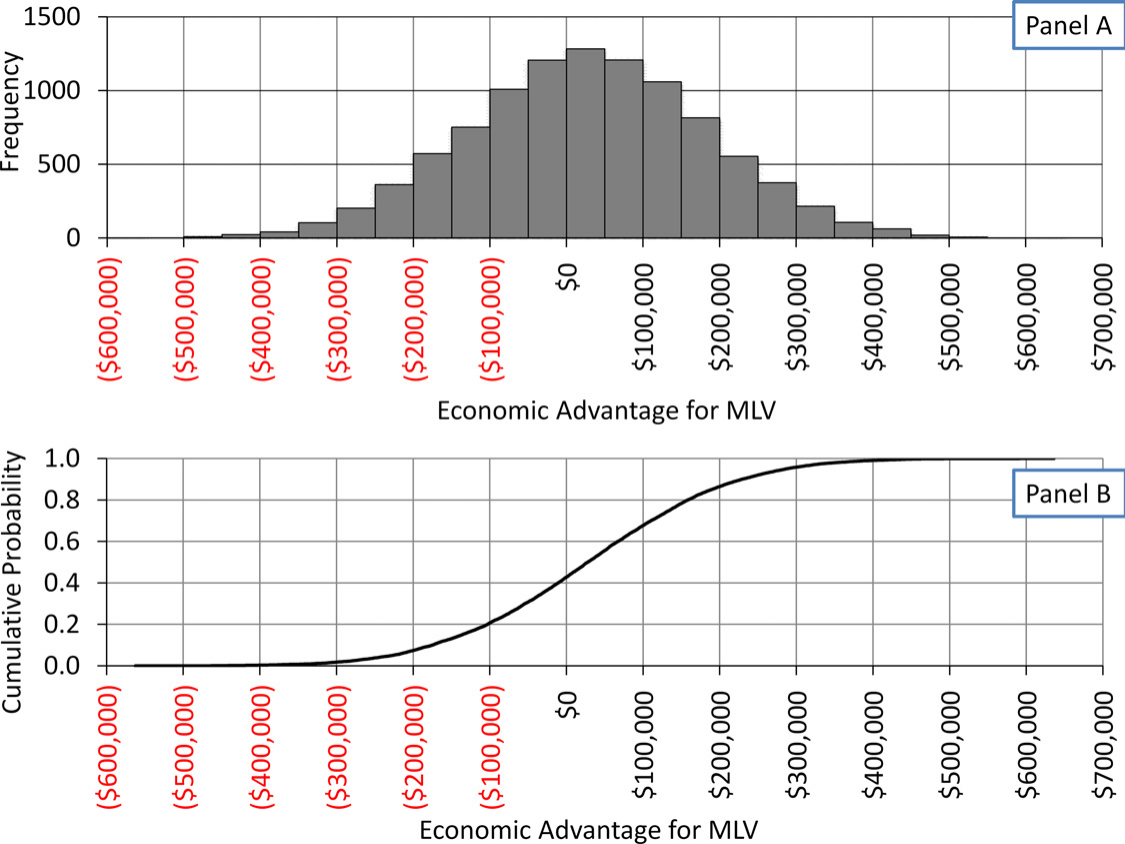
Distribution of economic advantage of farms that used MLV in comparison to those that used LVI as part of load-close-expose program. Outcome of model B, a Monte Carlo simulation of economic advantage of MLV compared to LVI. (A) is the outcome illustrated as a probability density function. (B) is the outcome illustrated as cumulative density function.

**Fig 4:**
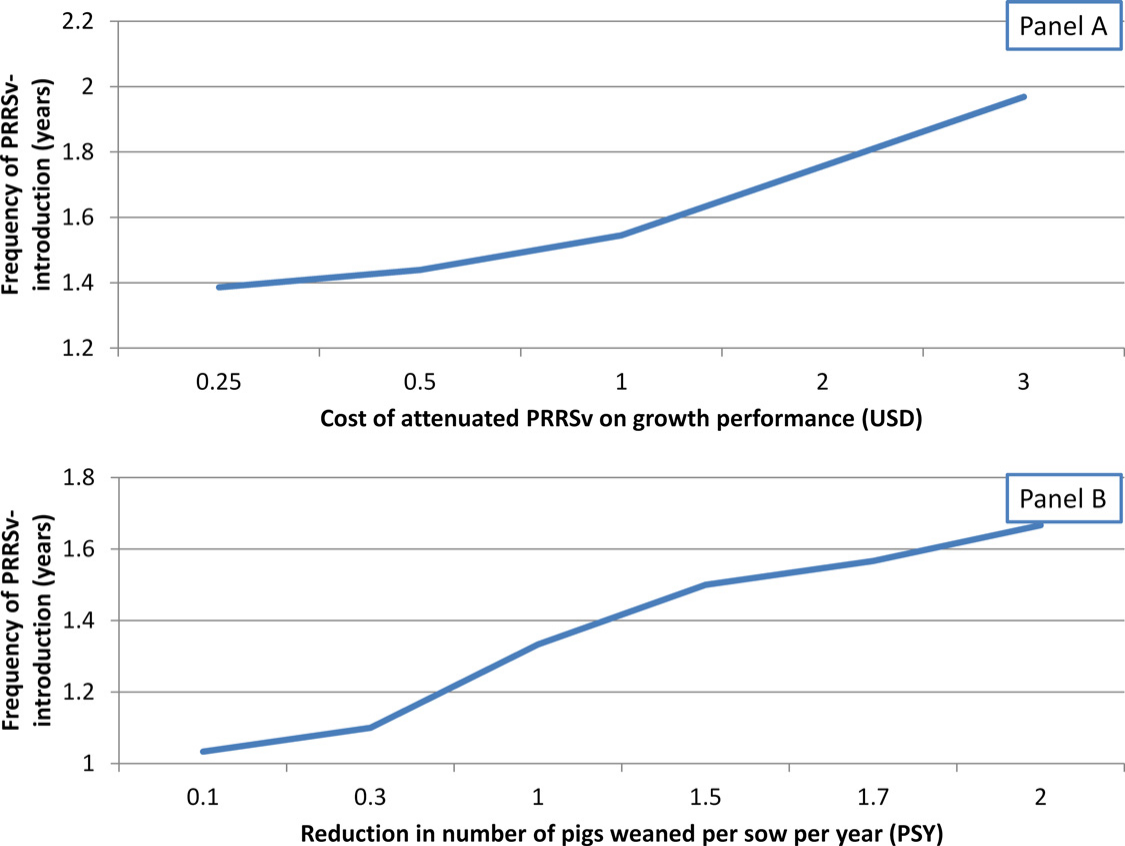
Break-even analysis of preventative vaccination practice according to cost of attenuated-PRRSv on growth performance or magnitude of reduction on pigs/sow/year (PSY) due to attenuated PRRSv. **(A).** Effect of attenuated-PRRSv impact on pig growth performance on break-even of preventative vaccination, considering sow herd-level impact of 1 PSY. (B) Effect of attenuated-PRRSv impact on reduction of pigs weaned/sow/year on break-even of preventative vaccination, assuming no impact of attenuated PRRSv on growth performance.

**Fig 5:**
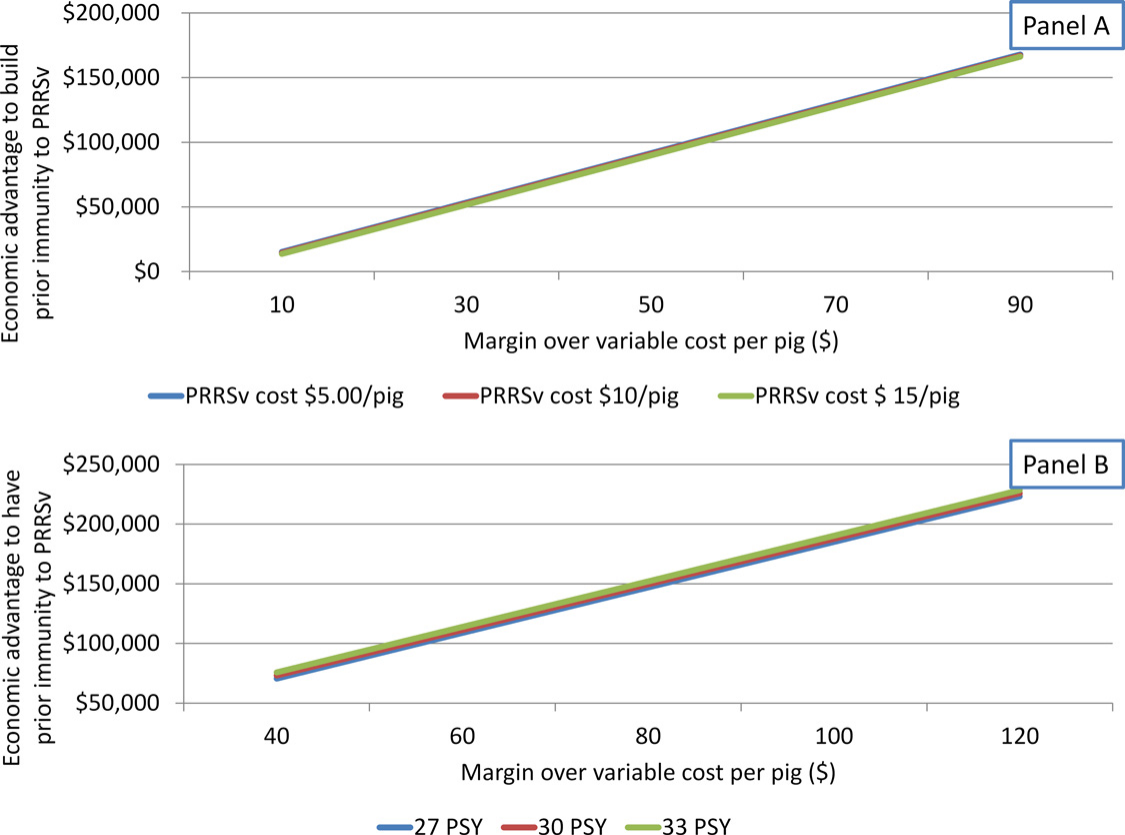
Break-even analysis of preventive vaccination practice according to cost of attenuated-PRRSv on growth performance or magnitude of reduction on pigs/sow/year (PSY) due to attenuated PRRSv. (A) Impact of margin over variable cost (MOVC) and production cost attributable to PRRSv on advantage to have prior PRRSv-immunity. (B) Impact of MOVC and PSY on advantage to have prior PRRSv-immunity.
